# Chitosan Nanoparticles as Oral Drug Carriers

**DOI:** 10.3390/ijms24054289

**Published:** 2023-02-21

**Authors:** Omar Rodrigo Guadarrama-Escobar, Pablo Serrano-Castañeda, Ericka Anguiano-Almazán, Alma Vázquez-Durán, Ma. Concepción Peña-Juárez, Ricardo Vera-Graziano, Miriam Isabel Morales-Florido, Betsabe Rodriguez-Perez, Isabel Marlen Rodriguez-Cruz, Jorge Esteban Miranda-Calderón, José Juan Escobar-Chávez

**Affiliations:** 1 Unidad de Investigación Multidisciplinaria-Lab 12, Facultad de Estudios Superiores Cuautitlán-Universidad Nacional Autónoma de México, Carretera Cuautitlán-Teoloyucan, km 2.5 San Sebastián Xhala, Cuautitlán Izcalli 54714, Mexico; 2 Unidad de Investigación Multidisciplinaria L14 (Ciencia y Tecnología de los Materiales), Facultad de Estudios superiores Cuautitlán, Universidad Nacional Autónoma de México, Estado de México 54714, Mexico; 3 Instituto de Investigaciones en Materiales, Universidad Nacional Autónoma de México, Apartado Postal 70-360, CU, Coyoacán, Ciudad de México 04510, Mexico; 4 Laboratorio de Farmacia Molecular y Liberación Controlada, Departamento de Sistemas Biológicos, Universidad Autónoma Metropolitana, Xochimilco 04960, Mexico; 5 Laboratorio de Servicio de Análisis de Propóleos (LASAP), Unidad de Investigación Multidisciplinaria (UIM), Facultad de Estudios Superiores Cuautitlán, Universidad Nacional Autónoma de México, Cuautitlán Izcalli 54714, Mexico; 6 Unidad de Enseñanza e Investigación, Hospital Regional de Alta Especialidad de Zumpango, Carretera Zumpango-Jilotzingo #400, Barrio de Santiago, 2ª Sección, Zumpango 55600, Mexico

**Keywords:** chitosan, chitosan nanoparticles, oral administration

## Abstract

The use of nanoparticles as drug delivery systems has increased in importance in the last decades. Despite the disadvantages of difficulty swallowing, gastric irritation, low solubility, and poor bioavailability, oral administration stands out as the most widely used route for therapeutic treatments, though it may not always be the most effective route. The effect of the first hepatic pass is one of the primary challenges that drugs must overcome to carry out their therapeutic effect. For these reasons, controlled-release systems based on nanoparticles synthesized from biodegradable natural polymers have been reported to be very efficient in enhancing oral delivery in multiple studies. Chitosan has been shown to have an extensive variability of properties and roles in the pharmaceutical and health fields; of its most important properties are the ability to encapsulate and transport drugs within the body and enhance the drug interaction with the target cells, which improves the efficacy of the encapsulated drugs. The physicochemical properties of chitosan give it the ability to form nanoparticles through multiple mechanisms, which will be addressed in this article. The present review article focuses on highlighting the applications of chitosan nanoparticles for oral drug delivery.

## 1. Introduction

During the last decades, alternative ways of medication administration have gained attention. Several delivery route options are outlined in this review, along with their strengths and weaknesses; fundamental and physicochemical criteria features that would make a drug an appropriate candidate for pharmaceutical formulation; and methods to evaluate delivery viability, toxicity at the place of delivery, and feasibility [1].

There are numerous studies on novel drug delivery approaches, but oral administration remains the most effective and easiest to administer, and it induces minimal side effects [1,2]. However, the main disadvantage of oral administration is poor bioavailability [3]. To overcome these limitations, the use of nanocarriers as drug delivery systems by oral route has become known, thanks to the development of nanotechnology and, more specifically, nanomedicine.

Nanomedicine is a subdivision of nanotechnology, which uses nanometric particles [4]. Nanoparticles (NPs) are capable of functioning as pharmaceutical carriers for a variety of delivery systems. Studies have shown that NPs have been applicable for use in the pharmaceutical and biomedical sectors to treat illnesses including diabetes, cancer, and HIV [5]. Furthermore, NPs can interact with the immune system in many ways, particularly by eliciting inflammation and interacting with dendritic cells. In addition, NPs have been developed to increase therapeutic limitations and membrane crossing, and with the development of personalized therapies, their therapeutic efficacy has been improved [6].

NPs can be comprised of a multitude of substances, but some existing NPs may be toxic to humans [5]. Polymeric NPs, such as the chitosan NP, are commonly 10–1000 nm in dimension and, being formulated from polymers, have a natural bioadaptability, biocompatibility, and biodegradability [4].

Chitosan NPs have the benefit of slowing and controlling the release of drugs, improving their solubility and stability, and decreasing their toxicity [7].

This review focuses on the development, importance, and impact that chitosan-based polymer NPs have acquired in the fields of pharmacology and health, highlighting the novel alternatives for drug delivery, the reduction in adverse effects, and the upgrading of bioavailability, efficacy, and acceptance by patients.

## 2. Drug Administration

Drug administration depends on the individual’s physiology and the formulation [8].

The absorption mechanism and characteristics of the drug are the essential aspects that define the proper delivery system for optimal bioavailability and efficacy. In contrast to drug prescription, which mostly lies in the hands of health care personnel, drug administration is an everyday practice for almost all humans [9,10].

### Drug Delivery Systems

Drug delivery systems are engineered technologies that perform the targeted delivery and/or controlled release of therapeutic agents [11,12].

Many of the pharmacologic properties of free drugs can be improved using drug delivery systems such as nanocarriers, which are made primarily of lipids or polymers and their associated therapeutics [11].

Drug delivery systems are designed to either alter the pharmacokinetics (PK) and biodistribution (BD) of their associated drugs, function as drug reservoirs (i.e., as sustained-release systems), or can sometimes perform both functionalities.

Nanotechnology is a novel pharmaceutical technology pertaining to nanosized particles of a variety of materials, and it opens a new avenue for drug delivery methods. According to the U.S. Environmental Protection Agency (EPA), nanotechnology is defined as “the creation and use of structures, devices, and systems that have novel properties and functions because of their small size” [12,13].

## 3. Non-Oral Administration Routes

In comparison with the oral route, the intramuscular route avoids the gastrointestinal tract. However, it presents pain and risk of injury, and it requires adequate personnel (Table 1) [9].

Other administration routes, such as ocular, transdermal, subcutaneous, or nasal delivery, have also been developed for localized drug administration with the avoidance of undesirable systemic effects [11].

The aim of the transdermal route is to deliver the medication across the skin layers to the blood tissue. Drug absorption in this case occurs through the intercellular, transcellular, and transappendageal pathways [9,11]. Transdermal drug delivery systems present many inconveniences, including skin irritation or contact dermatitis, risk of allergic reactions, poor permeability of some drugs through the skin, and insufficient skin absorption of drugs with large particle sizes [12]. Possible toxic effects and drug uptake limitation are important in transdermal systems as skin conditions change with age [13].

Subcutaneous drug administration is used due to the simplicity of the injection method, the ability to deposit large volumes of medication, and the freedom of choosing a specific injection site. However, with this administration route, the rate and extent of bioavailability are dependent on a large number of biopharmaceutical and biological factors [14].

An alternative route that presents significant challenges is the ocular drug delivery route. The anatomy, physiology, and biochemistry of the eye render this organ highly impervious to external substances. In ocular drug delivery systems, the primary limitation is the rapid and extensive elimination of conventional eye drops, resulting in extensive loss of the medication. Only 5%–10% of the total administered medication reaches the target tissue, causing an extremely poor intraocular bioavailability [15].

Inhaled medications are one of the cornerstones of pharmacological treatment. Several inhaler devices exist, and each device has particular attributes to get the optimal inhalation of drugs; however, the correct use of the inhaler device is not guaranteed and is prone to patient error. Moreover, eliminating the toxicity of NPs, polymers, and other excipients is critical for the development of safe inhalable formulations [16,17].

## 4. Oral Administration

Despite some disadvantages, oral administration is the natural route of drug administration, which has advantages (Table 2) such as sustained release, ease of administration, and ease of use. In addition, the GI tract’s large surface area (>300 m^2^) lined with a mucosal layer paves the way for drug attachment and subsequent absorption [9,18]. Many developments have been made to optimize oral drug absorption, including the use of absorption enhancers, enzyme inhibitors, enteric coating, and microparticles or nanoparticles [19].

The main function of the GI tract is food digestion and protection against microbial agents [20].

The oral administration of drugs is desirable, but there occurs the enzyme degradation of these drugs, and of the drug compounds (Table 3) [21]. Oral ingestion remains the preferred mode of delivery for most drugs, largely due to simplicity [22].

The gastrointestinal tract has an area of 300–400 m^2^ for drug absorption by enterocytes, and the gastrointestinal tract contains specialized cells such as Peyer cells, M cells, and goblet cells for this function [19]. In the GI tract, any drug will encounter a series of barriers before it reaches the capillaries in the subepithelial tissue [20].

### 4.1. Gastrointestinal Tract Structure

The function of the GI tract includes digestion, excretion, and protection. The GI tract has many sophisticated and autonomous functions coordinated over a range of length and time scales [23]. The GI tract can be divided into upper and lower portions. The oral cavity, pharynx, esophagus, stomach, and the initial portion of the small intestine, known as the duodenum, are the parts that make up the upper GI; on the other hand, the lower GI tract includes the rest of the small intestine, consisting of the jejunum and ileum, as well as the large intestine, consisting of the cecum, colon, and rectum. The structure of the GI tract is similar throughout all its segments [9].

The GI tract developed to enable the transport of nutrients throughout its length. The small intestine measures approximately 1.5 m in length, with a diameter of 6–7.5 cm. The surface area of the small intestine is significantly enlarged by the existence of villi and microvilli, which increase the intestinal surface area 30-fold and 600-fold, respectively (Table 4). Furthermore, drug molecules trapped within the GI tract mucus are protected against the shearing forces caused by flowing gastric juice [9,24]. Absorption is helped by the increased mucosal surface area provided by elongated villus folds lined by absorptive enterocytes. Each enterocyte has microvilli that comprise a fine apical brush border that further increases surface area [25].

Intestinal mucus is the main barrier through which ingested NPs must pass. Surface charge can play a critical role in absorption. A net neutral or positive surface charge prevents mucoadhesion and allows penetration, while a negative surface charge in hydrophilic and lipophilic compounds hinders penetration. Small NPs penetrate easier than bigger ones [25].

The small intestine is the main site of nutrient absorption. The pH of the duodenum is 6–7 in humans, 4 in mice and 5 in rats. The physicochemical behavior of the intestine is complex.

The intestinal epithelium is the major barrier limiting the absorption of macromolecules [26]. This epithelial cell layer is made of enterocytes, goblets cells, and M cells. The most abundant in the intestine is the enterocyte cells, which specialize in transporting nutrients by active transport or passive diffusion [27].

### 4.2. Devices and Materials

The oral administration of drugs has limitations, such as low stability in the GI tract, as well as low permeability and solubility. That is the reason for the application of pharmaceutical biotechnology to improve the physicochemical and biopharmaceutical properties of pharmaceuticals [28].

The devices developed for oral drug administration can be classified as intestinal patches, GI microneedles, and particulate carriers, which include microparticles, NPs, micelles, and liposomes [9].

Swelling polymers increase their weight (10–1000 times) in aqueous media. Swelling polymers have been used to develop and generate swellable matrices or devices and super disintegrants [29].

Associated with chemical approaches, physical strategies are more feasible in improving the pharmacodynamic and pharmacokinetic properties of drugs. These approaches are based on covalent and noncovalent interactions of drugs with absorption enhancers, enzyme inhibitors, or colloidal carrier systems such as microparticles, NPs, or nanoemulsions [30].

Surfactants, chelating agents, and fatty acids are regular absorption enhancers that increase drug penetration due to their capacity to alter the membrane fluidity of the intestine’s lipid bilayer [31]. Enzyme inhibitors contribute to protecting peptides from degradation while in the GI tract. To improve the oral bioavailability of peptides and proteins in drugs, enzyme inhibitors are usually used in combination with absorption enhancers [32,33].

Enhanced oral delivery includes the improvement of the physicochemical properties of pharmaceuticals as well as nanocarriers [28].

Erosion of polymers is a complex phenomenon, as it involves swelling, diffusion, and dissolution. Erosion occurs in two ways: homogeneously and heterogeneously. Homogenous erosion occurs at the same rate throughout the matrix, whereas heterogeneous erosion occurs from the polymer’s surface toward its inner core [34].

## 5. Ingestion of Nanoparticles

The intake of exogenous, engineered nanoparticles primarily results from hand-to-mouth contact in the workplace. Nanoparticles can be ingested directly via food, drinking water, drugs, or drug delivery systems [35].

Both the biological characteristics of the GI tract as well as the properties (particle size, coating, aggregation, among others) of the NPs impact ingestion and bioavailability studies [25]. Another challenge in oral drug administration is short gut residence time and poor mucosal contact; a method to overcome this is the use of adhesion promoters such as linear or tethered polymer chains to promote bio-adhesion, which has been well documented [36].

The translocation of particles through the intestinal barrier is a multistep process that requires diffusion across the mucus layer, contact with enterocytes and M cells, and uptake via cellular entry or paracellular transport. The most common mechanism for the uptake of NPs into intestinal epithelial cells appears to be endocytosis [25].

One way to overcome absorption barriers is the generation of gene NPs. These protect the gene or drug and enhance cellular uptake through endocytosis, of which a promising polymer for these systems is chitosan [21].

One benefit of NPs’ formulation is the potential for providing targeted and localized drug delivery. Several studies have shown that NPs can increase the oral bioavailability of drugs through different mechanisms [22].

The generation of orally targeted NPs is essential to understand the disease as well as the physiological barriers and specific receptors presented by the different regions of the GI tract [22]. The convenience and other advantages of oral delivery also make NP formulation a promising strategy for mass vaccination programs [9].

## 6. Polymeric Nanoparticles

NPs as a delivery system have achieved advantages by overcoming the challenges of typical dosage methods [36]. According to the definition from the National Nanotechnology Initiative (NNI), NPs are structures with sizes ranging from 1 to 100 nm in at least one dimension. However, the prefix “nano” is commonly used for particles that are up to several hundred nanometers in size [37].

**Figure 1:**
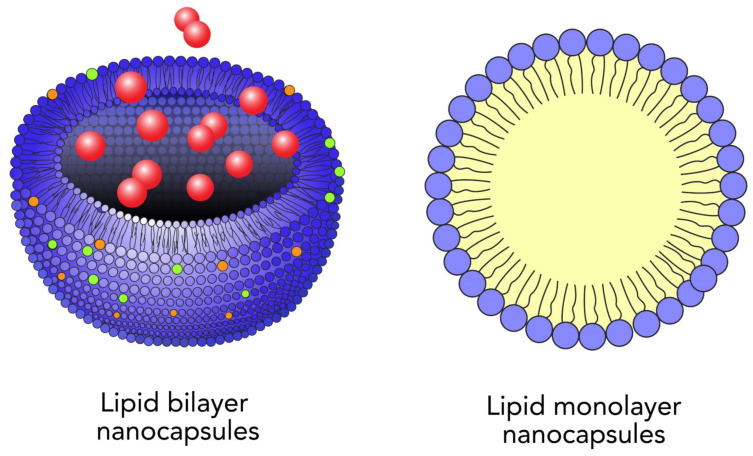
Example of mono and bilayer nanoparticles.

Nanotechnology is the science of the nanoscale [38]. NPs are made up of three layers: (a) the surface layer, (b) the outer layer, and (c) the core (Figure 1) [39].

The nanocarriers must be able to integrate into the biological system and must not cause any negative or toxic effects [40].

NPs have also been used in immunotherapy (vaccines), which, by containing foreign substances and being in contact with the immune system, generates a specific immune response. Properties such as size, charge, and rigidity, among others, determine their interaction with the immune system [41].

Polymeric NPs can deliver drugs and overcome biological barriers, as well as target drugs to specific cells [42].

Most polymeric NPs are now biodegradable and biocompatible due to the accomplishments of researchers in the last few decades in developing NPs for drug delivery systems. These biodegradable NPs are coated with controlled-release polymers that can release proteins, peptides, antigens, DNA transporters, and target specific sites [43].

Polymeric systems have become popular due to their ability to provide a sustained release of the associated active compounds [44]. For more than five decades, the pharmaceutical industry has been using procedures such as coating and encapsulation to incorporate polymers with bioactivity [45]. Various polymers have been used for site-specific drug delivery while minimizing side effects [46].

Biodegradable and bioerodible polymers are a crucial group of materials for drug delivery [47]. Biopolymers are of natural origin (vegetable, animal, bacterial, fungi). They include networks of polysaccharides, cellulose, starch, gelatin, and collagen among others, with potential applications in the pharmaceutical industry [48]. In recent years, biodegradable polymers have garnered considerable attention as potential drug delivery devices, given their applications in the controlled release (CR) of drugs, their ability to target particular organs and tissues, their potential as carriers of DNA in gene therapy, and their ability to deliver proteins, peptides, and genes through a peroral route of administration [46].

The objective of a delivery system is to release at the desired site for a specified time to exert the therapeutic effect [45].

The purpose of nanotechnology is the delivery drugs to target sites so that the pharmacologically desired effect of the drug is maximized and the limitations and drawbacks that would hinder the required effectiveness are overcome [49]. NPs consist of macromolecular materials and can be used therapeutically as adjuvants in vaccines or drug carriers [50]. The challenges of biological barriers such as the passage of substances through the blood-brain barrier have been overcome with the use of NPs [49].

**Figure 2:**
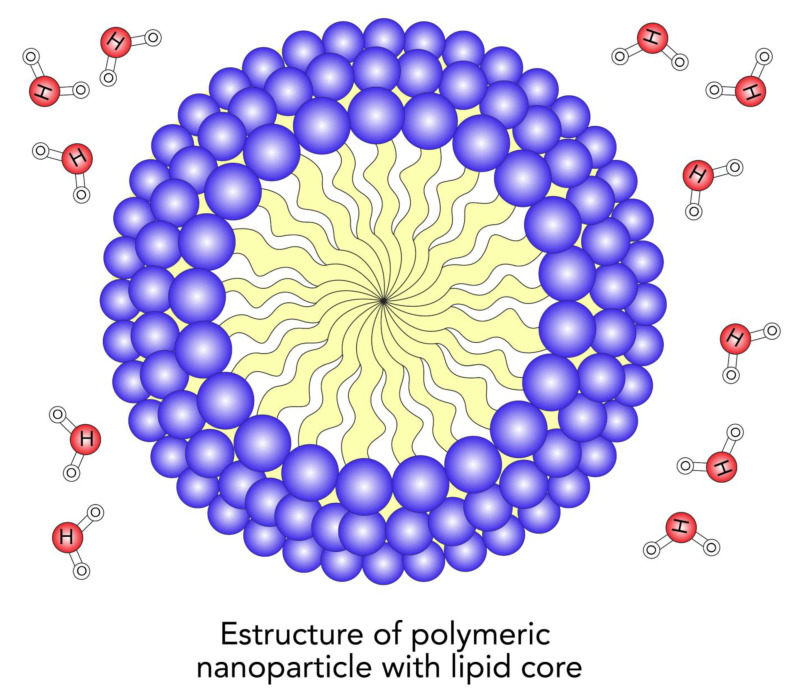
Nanoliposomes.

Polymeric NPs, which possess better reproducibility profiles than liposomes (Figure 2), have been used as alternative drug carriers to overcome many drug delivery problems. Polymers used to form NPs can be either synthetic or natural polymers [50]. Natural polymers such as chitosan, albumin, and heparin have been used for the delivery of oligonucleotides, DNA, and proteins, as well as drugs. Polymeric NPs are popular due to their ability to deliver drugs, as well as for their biodegradability. Chitosan is one of the most widely used cationic polymers [51].

Environmentally responsible polymers are a class of smart polymers consisting of linear, cross-linked copolymers (Table 5). Their characteristic feature is their ability to undergo physicochemical change in response to external stimuli such as pH, temperature, etc. [45].

Polymeric microparticles and NPs have been applied to gene delivery, and particularly in vaccine design (e.g., DNA vaccine). Synthetic vectors based on polycation enable gene delivery by cell-targeted ligands [51].

**Figure 3:**
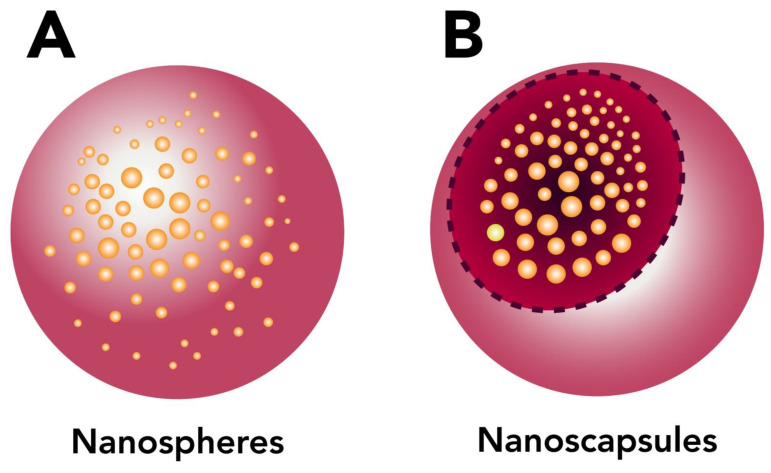
(**A**) Scheme of nanospheres where the active is dispersed throughout the particle. (**B**) Scheme of nanocapsules where the active is in the core.

There are two varieties of NPs, depending on the preparation process: nanospheres or nanocapsules (Figure 3) [55]. Nanospheres have a matrix structure where the drug is located. The nanocapsules have a membrane and contain the drug inside [50].

### 6.1. Preparation of Polymeric Nanoparticles

Polymeric NPs have been widely explored in the pharmaceutical fields since their launch [56,57]. Polymeric NPs can be prepared by different methods, such as solvent evaporation, nanoprecipitation, salting out, dialysis, and supercritical fluid technology [43].

Among the available families of nanocarriers, polymeric vectors have been widely researched, owing to several beneficial properties, including biocompatibility, biodegradability, non-immunogenicity, and nontoxicity. Progress has also been made in designing potent, stable NPs for tissue and cell targeting by conjugating ligands in the polymeric NPs. Altogether, these advancements have improved NP performance [56].

The synthesis of metal NPs includes the processes of spray pyrolysis, liquid infiltration, rapid solidification, and others. The synthesis of ceramic nanocomposites includes the powder process, polymeric precursor process, and sol-gel process. Finally, the fabrication of polymeric nanocomposites includes intercalation, in situ intercalative polymerization, melt intercalation, template synthesis, mixing, in situ polymerization, and the sol-gel process [58].

#### 6.1.1. Ionic Cross-Link Method

**Figure 4:**
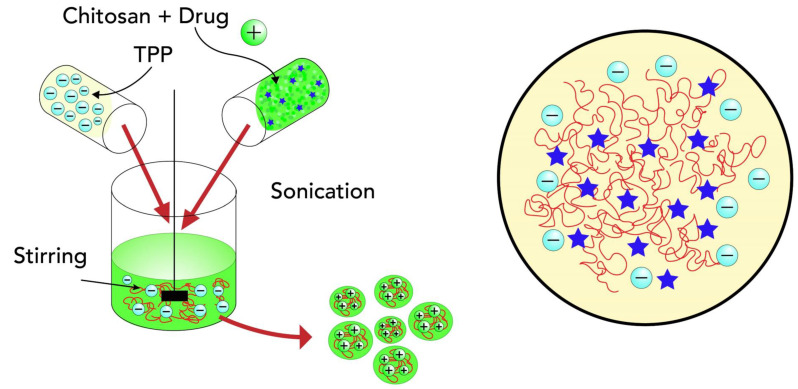
Synthesis of nanoparticles based on the ionic crosslink method.

In this technique, an ionic cross-link is conducted by the aggregation of chitosan or its derivates with oppositely charged macromolecules or in the presence of an ionic cross-link agent (Figure 4) [59].

A cross-link is formed through a chemical reaction, such as van der Waals forces, which link two polymers together [60].

Ionically cross-linked chitosan NPs are based on the formation of complexes with the amino group and a polyanion (tripolyphosphate TPP) [61].

#### 6.1.2. Covalent Cross-Link Method

Covalent cross-linking is more attractive than the ionic gelation method. Bodnar et al. [61] reported that the synthesis of chitosan NPs covalently cross-linked with tartaric acid resulted in particles 60–280 nm in size [62]. Covalent cross-linking enhances the chemical and mechanical properties of the material [63].

#### 6.1.3. Reverse Micellar Method

In the reverse micellar formation of NPs, the surfactant is dissolved in an organic solvent forming micelles and chitosan is added under continuous stirring; to this transparent solution, a crosslinker is added. The limitations are the use of organic solvents as well as the washing steps [61].

#### 6.1.4. Precipitation/Coacervation

NPs are prepared by alkaline precipitation (pH > 6.5). Chitosan is injected into the organic solvent by employing a nozzle. The NPs are obtained by filtration or centrifugation, then rinsed with water, and a crosslinking agent is added to modulate the release of the substances [61].

#### 6.1.5. Emulsion–Droplet Coalescence Method

The NPs are formed by the emulsification of an organic polymeric solution in an aqueous phase, after which the organic solvent is evaporated. The organic solution is poured into the aqueous phase. Emulsification is carried out under high-shear force conditions to reduce the size of the emulsion droplet. The evaporation of the solvent leads to the formation of NPs [61].

## 7. Chitosan Nanoparticles

Chitosan is a cationic polysaccharide and has been considered a promising nanomaterial [64]. Chitosan offers outstanding biological properties, including biocompatibility, biodegradability, and nontoxicity, that make it increasingly important in various applications in the pharmaceutical and biomedical fields [65].

The shellfish industry makes very common use of the meat, and the head and shells are discarded as waste (80,000 tons of waste per year) [66]; shell waste is recycled to obtain commercially viable products such as chitin [67].

Chitosan is the N-acetyl derivate of chitin obtained by N-deacetylation. Chitosan is widely used in the encapsulation of active food ingredients, enzyme immobilization, controlled drug delivery, and plant growth promotion in agriculture. Chitosan has properties such as biodegradability, biocompatibility, antimicrobial, bioactivity, nontoxicity, and a polycationic nature [68].

**Figure 5:**
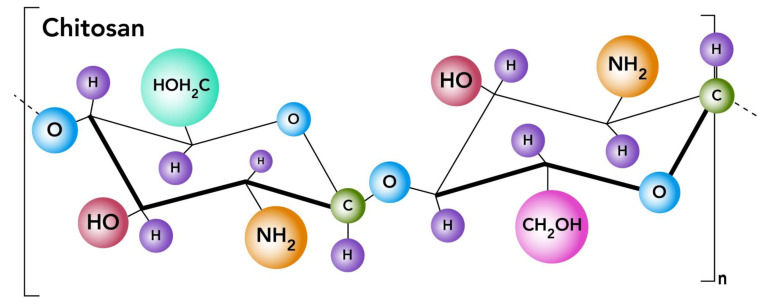
Chitosan molecular structure.

Chitosan (Figure 5) is well-known for its hydrophilic, biocompatible, biodegradable, and nontoxic properties. The use of chitosan NP for oral and nasal drug delivery routes has been reported in previous studies [69]. NP technology is an increasingly accepted formulation technique as it overcomes the limitations of conventional oral drug delivery.

The positively charged chitosan will bind to cell membranes and is reported to decrease the trans-epithelial electrical resistance (TEER) of cell monolayers, as well as to increase paracellular permeability. Chitosan solutions have been shown to increase transcellular and paracellular permeability in a reversible, dose-dependent manner that depends on the molecular weight and degree of deacetylation of the chitosan. Low-molecular-weight chitosan possesses the ability to penetrate cells, where it is suspected of binding to cell DNA, prohibiting mRNA synthesis and causing the termination of cell multiplication [60]. The mechanism of action, which includes interaction with the tight junction proteins and ZO-1 proteins, redistribution of F-actin, and slight destabilization of the plasma membrane, appears to be mediated by the positive charges on the chitosan. Thus, the ability of chitosan to enhance permeation is influenced by the pH of the environment [21].

Chitosan is biodegradable and, due to its low molecular weight, is eliminated by the kidneys, and if it is of higher molecular weight, it can be degraded into smaller fragments for renal elimination.

Mucoadhesive NPs are able to have their surface coated with mucoadhesive polymers such as chitosan or Carbopol [66]. Mucus is a blend of molecules including salts, lysozyme, and mucins, which are highly hydrated glycoproteins primarily responsible for the viscoelastic properties of mucus. Sialic acid residues on mucins have a pH of 2.6, making them negatively charged at physiological pH [21].

In addition, the formation of chitosan into microparticles and NPs also preserves mucoadhesion [21]. The application of biodegradable nanosystems is one of the most successful advancements in the pharmaceutical industry [40].

Chitosan can be used as an oral gene carrier due to its adhesive properties. On the other hand, researchers have found that in vitro, chitosan-mediated transfection depends on the cell type, serum concentration, pH, and chitosan molecular weight [51].

The protection offered by NPs has generated the development of systems with macromolecules and proteins, among others, since these promote the absorption of therapeutic substances [61].

**Figure 6:**
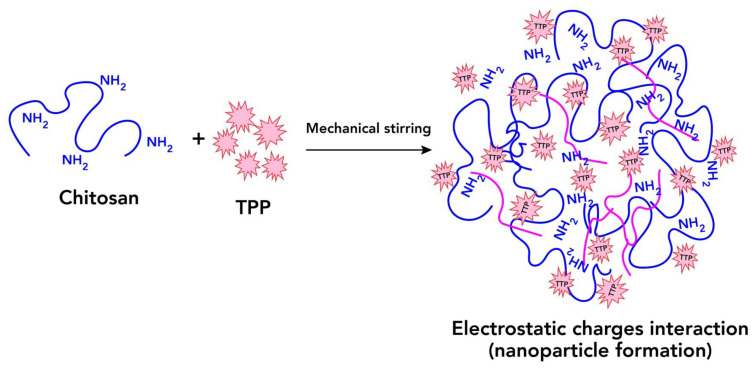
Electrostatic interaction between NH2 group of chitosan and negative charge of TPP.

The process of NP formation is based on electrostatic interactions between the amine group of chitosan and a negatively charged group of polyanions such as tripolyphosphate (Figure 6). This method is easy in aqueous media [51].

One study has shown that the intratumoral administration of interleukine-12 co-formulated with the biodegradable polysaccharide chitosan could enhance the anti-tumor activity of interleukine-12 in mice bearing established colorectal (MC32a) and pancreatic (Panc02) tumors [51].

Chitosan nanospheres have applications for drug delivery in the gastrointestinal, ophthalmic, nasal, sublingual, transdermal, and vaginal tract [51].

The absorption-promoting effect of chitosan has been extensively studied by the combination of mucoadhesion and the transient opening of tight junctions in the mucosal cell membrane, which have been experimentally verified both in vitro and in vivo [61].

The mechanism of chitosan NP transport across the GI tract is likely through adsorptive endocytosis. Chitosan NP internalization is higher in the jejunum and ileum than in the duodenum [61].

Chitosan NPs can be applied to mucosal delivery (pulmonary, nasal), where peptides and proteins can be administered [66].

Oral formulations are considered a desirable alternative to intravenous drug administration due to the advantage of offering adaptability to tune the dosing schedule to individual patient responses based on efficacy and toxicity. Oral formulations of NPs can increase the number of patients treated [70].

### 7.1. Applications of Chitosan Nanoparticles for Oral Drug Delivery

Recently, the use of chitosan in pharmaceutical development has increased due to its compatibility with other components such as surfactants, starches, etc. Chitosan increases cell membrane permeability, both in vivo and in vitro. Chitosan has the potential of serving as an absorption enhancer across intestinal epithelia, prolonging the residence time of delivery systems at absorption sites and relaxing the tight junctions of cell membranes [71]. The cationic nature of chitosan permits it to form complexes with oppositely charged drugs and excipients, thereby altering the physicochemical characteristics of the formulation. Reacting chitosan with controlled amounts of multivalent anions results in cross-links between chitosan molecules (Table 6) [72].

#### 7.1.1. Gene Delivery

In gene therapy, transfection is hindered by the orientation of the system to the target cell as well as the degradation of endolysosomes and intercellular trafficking of plasmid DNA [95].

Gene carriers have the disadvantage of low transfection and toxicity, and they even provoke severe immune responses [7]. Nucleic acids are being developed for gene therapy and vaccination [21]. As a non-virus carrier, chitosan has exceptional compatibility and biodegradability [7,95].

Dastan and Turan [96] improved chitosan microparticles and reported a sustained release profile of DNA with a high potential transfer of DNA tested in different cell lines such as human embryonic kidney, Swiss 3T3, and HeLa [97].

#### 7.1.2. Protein and Peptide Delivery

Proteins and peptides usually have a high molecular weight and low lipophilicity, which is why they are usually administered subcutaneously. However, NPs have been shown to administer peptides and proteins orally. Chitosan NPs are gaining increased attention for their ability to serve as carriers for oral protein and peptide delivery [80,87].

#### 7.1.3. Drugs

Antiviral, antiallergic, and hormone drugs can be loaded in chitosan NPs through an ionic cross-link method [7,98]. For example, in the research performed by Shailender et al. [89], the ionic gelation method was used in the preparation of tenofovir disoproxil fumarate chitosan NPs [50,99].

Cancer has become one of the most lethal and prevalent conditions throughout the world. The success of current therapies is primarily limited by tumor recurrence, metastasis, acquired resistance, and the presence of side effects [100]. The cancer treatment doxorubicin, for example, produces side effects such as cardiotoxicity. To minimize these side effects, the drug has been encapsulated in chitosan NPs. This has caused several advantages, including better delivery, improved cell- or tissue-targeted drug delivery, and enhanced absorption in the entire small intestine [101,102].

Tuberculosis continues to be the leading cause of mortality worldwide, and it is also an occupational disease in health care. Noncompliance is the primary limitation of treatment, largely because treatment involves continuous, frequent, and multidrug dosing. Chitosan NPs could improve a long-duration drug formulation, releasing the antitubercular agents in a slow and sustained manner [92].

#### 7.1.4. Vaccines

The effect of chitosan as an adjuvant for the generation of vaccines makes it a safer therapy [71].

Oral vaccination is a highly promising application of chitosan NPs. Food allergy is a common and often fatal condition with no effective treatment. Orally administering NPs prepared by complexing plasmid DNA with chitosan has been shown to result in a transduced gene expression in the intestinal epithelium of patients with food allergies [103].

Olivera et al. [104] developed a vaccine for the control of schistosomiasis, which is recognized as the most important human helminth infection in terms of morbidity and mortality. They described that chitosan NPs with plasmid DNA encoding the Rho1-GTPase protein of *Schistosoma mansoni* were able to induce high levels of the modulatory cytokine IL-10. It resulted in a significant reduction in liver pathology. Mice immunized with only chitosan NPs presented with 47% protection against parasitic infection.

## 8. Discussion

The oral route of administration continues to be the first choice of both patients and doctors; although, as mentioned, the administered medications must cross many barriers and physiological processes that reduce their bioavailability and, consequently, their efficacy. For this reason, nanoparticulate or nanometric drug delivery systems based on biocompatible polymers have acquired great relevance. Their physicochemical characteristics have allowed them to be included within the unconventional forms of administration, and they have enabled the vectorization of active ingredients with low solubility or bioavailability, increasing their interaction with the target organs or cells through different routes of administration. Due to their easy preparation and high capacity to encapsulate peptides, drugs, and genes without interfering with their biological activity, chitosan-based NPs are the first choice when treating diseases through non-conventional methods. Chitosan NPs enable medications to cross physical and biological barriers, increasing bioavailability and leading to a more powerful effect with fewer adverse effects, which can be achieved without requiring invasive or painful routes of administration by using the oral route.

## Figures and Tables

**Table 1:** Advantages and disadvantages of oral administration vs. other routes.

	Administration Pathway	Advantages	Disadvantages
Enteral	Oral	Presents the greatest comfort for the patient and the most economical. The effect can be local or systemic Easy access. Formulations can be created that protect the active ingredients from degradation.	Impossible to use in uncooperative patients with vomiting or loss of consciousness. Unpredictable absorption due to the first pass liver effect. In the case of stomach or bowel surgery, it is not an option.
	Rectal	Excellent absorption through hemorrhoidal veins that connect directly into the vena cava. Prevents the effect of the first liver past.	It is an unpleasant way for patients. It cannot be used after surgery.
Parenteral	Subcutaneous	They are the first choice for active ingredients with low bioavailability in oral forms. Rapid absorption and effect. Depending on the formulation, they can have a prolonged effect.	Absorption becomes irregular in poorly perfused areas or organs. Small volumes. Irritating substances cannot be administered.
	Intramuscular	Absorption and immediate effect. The first choice for drugs with low bioavailability orally or when the GI tract is compromised.	Painful A bad application can imply paralysis or muscular atrophy.
	Intravenous	Dependable and reproducible effects. The full dose enters the systemic circulation, allowing an immediate response.	Requires special equipment or trained personnel. It is invasive and painful. The devices as well as the contact area are susceptible to infection. There is no way to withdraw the drug in case of adverse reactions.
	Topic	Non-invasive and easy to administer. Painless. Local effect. Patient satisfaction levels are high.	Molecular way and low-fat solubility are determining factors for its absorption. Low absorption. Skin irritation.
	Inhale	Immediate absorption thanks to the large surface area of the respiratory tracts.	Bioavailability depends on the patient’s inhalation technique and the size of the molecule.

**Table 2:** Advantages and disadvantages of oral administration.

Advantages	Disadvantages
Simple to use and easy to access; it is convenient for the patient.	Patient cooperation is required.
It is a safe and practical way.	Absorption cannot be predicted due to irregular release.
In case of adverse reaction, it can be removed by physical means such as vomiting or gastric lavage.	Gastric mucosa irritation.
It is an economical and effective method.	The taste of some medicines can be unpleasant.
Does not require trained personnel or training.	In extremely apprehensive patient it is not useful.
The effect can be prolonged beyond the therapeutic period.	The effect can be prolonged beyond the therapeutic period.

**Table 3:** Challenges/barriers for oral drug delivery.

Physicochemical Barriers	Biopharmaceutical Barriers	Physiologic Barriers	Clinic Barriers
Low solubility.	Low permeability.	Presence of mucus.	Fed- and fasted-state variability in drug absorption.
pH dependent solubility or degradation.	Degradation in the GI tract.	Differences in the pH of the GI tract (pH 1.2–7.4).	Inter and intra individual differences in the oral route.
Extensive ionization at GI pH range.	Presence of drug efflux transporters.	Rapid gastric emptying.	Deficiencies in the permanence and emptying of the GI tract.
High lipophilicity.	Variation of pH and mucosal layer thickness variations in the GI tract, depending upon the location.	Gastric and intestinal motility.	Presence of diseases.
High molecular weight.		Effect of the firs live pass.	
		Presence of digestive enzymes and microbiota of the GI tract.	

**Table 4:** Physicochemical attributes of several sections of the GI tract.

Part	pH	Extent (cm)	Mean Length (cm)	Mucus Regular Depth (µm)	Mucus Income (hour)	Surface Area (m^2^)
Stomach	1.5–5	20	NA	245 +/− 200	24–48	0.053
Duodenum	5.0–7.0	17–56	680	15.5		252
Jejunum	6.0–7.4	280–1000				
Ileum						
Colon	5.5–7.0	80–313	93	132 +/− 25		0.35
Total			835			

**Table 5:** Criteria for ideal polymeric carriers for nanoparticle delivery systems [50].

Carriers	Nanoparticles
Raw materials for massive production [52]	Raw materials for massive production [52]
Low cost	Limited or no use of organic solvents
Reduction of environmental cost [52]	Reproducibility and repeatability
Easy and flexible processing methods [52]	Protect drugs and other molecules with biological activity against the environment [53]
Non-toxic and immunogenic	Freeze-drying capacity
Soluble in water	Stable after administration
Lightweight, chemical stability, and elasticity	Biocompatibility, biodegradability and non-toxicity [54]
Protect drugs and molecules against the environment [53]	Bioavailability and therapeutic index [53]
	Potential use for controlled release [53]

**Table 6:** Many drugs loaded in chitosan nanoparticles with medical applications.

Drug	Function	Results	Autor	References
Pramipexole hydrochloride	Symptoms of Parkinson’s disease	Diameter 243 +/− 12–337 +/− 13 nm Zeta potential 23 +/− mV Entrapment efficiency 63%	S. Papadimitriou, D. Bikiaris, K. Avgoustakis, E. Karavas, and M. Georgarakis	[73]
5-fluorouracil Tamoxifen Doxorubicin hydrochloride	Chemotherapeutic	Diameter: 283.9 +/− 5.25 nm Zeta potential 45.3 +/− 3.23 mV Encapsulation efficiency 44.28 +/− 1.69%	M. A. Mohammed et al., L. Sun et al., J. G. Rosch et al.	[34,74,75]
Chatechin and apigallocatechin Quercetin	Flavonoids (antioxidants)	Diameter 110–335 nm Zeta potential 30 mV	M. A. Mohammed et al., A. Dube et al., A. I. Barbosa et al.,	[34,76,77]
Alendronate sodium	Osteoporosis treatment	Diameter 200 nm	M. A. Mohammed, J. T. M. Syeda, K. M. Wasan, and E. K. Wasan	[34]
Cyclosporin A	Immunosuppression	Diameter: 150 nm Zeta potential: +30 mV	M. H. El-Shabouri	[78]
Protein and gene delivery	Gene therapy	Diameter: 350 nm	C. Y. Wong et al., R. M. Saeed et al.	[79,80]
Insulin	Diabetes mellitus treatment	Diameter: 100–200 nm Encapsulation efficiency: 85%	F. Cui et al., L. Li et al.	[81,82,83,84]
Deferoxamine	Iron-chelating drug	Diameter: 150–400 nm	M. Lazaridou et al.	[85]
Aspirin Probucol	Treatment of restenosis (hypolipemic and antiplatelet agent)		H. Liu and J. He	[86]
Interferon-α	Cancer treatment and antiviral activity	Diameter 200 nm Entrapment efficiency 89%	C. C. Cánepa, J. C. Imperiale, C. A. Berini, M. Lewicki, A. Sosnik, and M. M. Biglione	[87]
Foscarnet Tenofovir disoproxil fumarate	Antiviral agent Antiretroviral therapy (HIV)	Diameter 450 nm Zeta potential 20/25 mV Drug loading 55%	E. Russo et al., J. Shailender et al.	[88,89]
Heparin	Anticoagulant properties (venous thrombosis, pulmonary embolisms)	Microemulsion method Diameter 146 +/− 33 nm Zeta potential 35 mV	W. Dong et al. W. Dong et al.	[71,90]
Puerarin	Treatment of coronary heart disease.	Diameter 126.28 nm PDI 0.122 Encapsulation rate 94.49%	J. Yan et al.	[91]
Rifampicin	Antibiotic	Diameter 221.9 nm Entrapment efficiency 44.17% Drug loading 42.96%	B. K. Patel, R. H. Parikh, and P. S. Aboti	[92]
Curcumin diglutaric acid (CG)	Prodrug of curcumin	Diameter 345 nm Zeta potential 22.1 mV	F. N. Sorasitthiyanukarn, C. Muangnoi, P. Ratnatilaka Na Bhuket, P. Rojsitthisak, and P. Rojsitthisak	[93]
Liraglutide	Diabetes treatment	Diameter 100 nm Loading efficiency 92.5% Loading capacity 54.16%	F. Shamekhi, E. Tamjid, and K. Khajeh	[94]

## Data Availability

Data sharing not applicable.
